# The Gordon Research Conference on Parkinson’s disease: just what the doctor ordered

**DOI:** 10.1038/npjparkd.2015.19

**Published:** 2015-10-22

**Authors:** Beth A Vernaleo

**Affiliations:** 1 The Parkinson’s Disease Foundation, New York, NY, USA

## Abstract

The period from June 28 to July 2, 2015 marked the inaugural Gordon Research Conference (GRC) on Parkinson’s Disease held at Colby Sawyer College in New London, NH, USA. There were 184 attendees from 19 countries, indicating a clear interest and need for this type of meeting in the field of Parkinson’s disease. The meeting, chaired by David Sulzer and co-chaired by Andrew Singleton, invited 33 speakers to cover eight topics on Parkinson’s disease. Fifty posters were presented, and of these, eight innovative posters were invited to give short talks at the end of each session. Discussion Leaders at the beginning of each session ensured that there was cohesion to the themes being discussed, providing a running dialog throughout the meeting. The general consensus was that the first GRC on Parkinson’s Disease filled an important need within the research community, and that there is great excitement and interest for the 2017 GRC on Parkinson’s Disease.

Since the inception of the Gordon Research Conferences in 1941, 881 research topics have been covered in physical, chemical, and biological sciences (the very first GRC was on High Temperature Corrosion). In all this time, there had not yet been a GRC dedicated to Parkinson’s disease (PD). This changed on June 28th with the first Gordon Research Conference on Parkinson’s Disease, held at Colby Sawyer College in New London, NH, USA. Despite initial concerns by the GRC staff that the meeting would not draw a sufficient crowd, there were 184 attendees from 19 countries, and scores more were turned away after the slots were filled. Clearly, there was a need for this meeting, and the PD research community has recognized this. The meeting was supported primarily by a grant from the Parkinson’s Disease Foundation with further support from the Gordon Research Conferences, NINDS, and Merck. Due to the GRC policy that attendees cannot report results outside of the meeting, to facilitate discussion and free exchange of ideas, much of which includes unpublished data, this meeting report is limited to the general themes and ideas explored.

Chaired by David Sulzer, PhD along with Andrew Singleton, PhD as co-chair, the meeting invited 33 speakers to present cutting-edge data on eight areas of Parkinson’s disease research: neuronal selectivity and transmission of pathology, gene identification, dominant gene functions, recessive gene functions, disease mechanisms in organelle transport and turnover, aging processes and neurodegeneration, pathological synaptic circuitry, and compensatory synaptic mechanisms. Although these topics did not cover the entire breadth of PD research, as was acknowledged at the beginning of the meeting, the Chairs expressed hope that future meetings would explore other important aspects of PD research. The Chairs chose topics and speakers at the forefront of research in which conclusions had not been reached, with the explicit goal to generate discussion around areas of controversy.

The conference began with the discussion leader, Stanley Fahn, MD, providing a thorough clinical description of Parkinson’s disease, and a history of levodopa that is still the gold standard therapy for PD. Next, James Surmeier, PhD, gave the keynote address entitled, ‘From Determinants of Selective Neuronal Vulnerability to Possible Neuroprotective Therapy.’ He discussed the biological mechanisms underlying the STEADY-PD III trial, a Phase III, 56-center, 5-year trial on isradipine, in 300 *de novo* PD patients. Isradipine, an L-type Ca^2+^ channel antagonist used to control hypertension, may be protective in PD by lowering Ca^2+^ entry into neurons, and reducing mitochondrial oxidative stress. This first session provided a roadmap of how the basic science underlying Parkinson’s disease can be translated into new therapeutic strategies. This was a perfect way to kick off a basic science meeting focused on disease, and set the stage for the days to follow.

Over the next 4 days, a more complete picture of PD began to emerge, as scientists learned that their individual areas of research are more connected, often via protein interactions, than previously thought. Alpha-synuclein was a prominent topic; its structure, function, pathological transmission, and interactions with other proteins including the retromer complex and TOM20 were discussed. LRRK2 and its role in protein expression and neural transmission were also intensively covered, as were the roadblocks to using LRRK2 inhibitors as therapy owing to potential kidney effects. The role of the retromer complex in PD pathogenesis, which includes the newly identified PD gene *VPS35*, was covered in several talks. A healthy dose of PINK1, parkin, and their role in mitochondrial quality control and motility was also presented. Cellular recycling pathways were another hot topic. The normal role of autophagy, and how it is affected in PD provided greater perspective as to how damage in a single protein can cause widespread dysfunction in multiple cellular pathways. The final day of the conference took a step back to examine the bigger picture by focusing on pathological changes in brain circuitry that occur in PD and resulting clinical features such as dyskinesia and impaired locomotion, as studied in animal models of PD.

Although the science was intense, there was time set aside for activities that allowed attendees to interact, network, and start new collaborations—an important feature of any GRC. For young investigators, who were in abundance as both attendees and speakers, these opportunities were invaluable. They were able to gain feedback on their research, establish themselves as independent scientists, and form lasting relationships with established scientists in the field whose work they had only read about. Attendees were provided opportunities to take advantage of New Hampshire’s beauty by taking part in hikes, paddle boarding, and other outdoor activities. For those of us who prefer to avoid the sun, Dr Fahn and Lucien Côté, MD, gave a fascinating workshop on the history of PD that included their first-hand experience as clinicians from the advent of levodopa therapy, while Mahlon Delong, MD; Charles Wilson, PhD; and Thomas Wichmann, MD presented a workshop on the history of elucidating basal ganglia function, a topic in which they have been central players.

Entertainment was provided in the evenings, and the clear highlight was a riveting and hyperkinetic performance and workshop by outstanding Spanish flamenco dancers and musicians (Pedro Cortes, Sonia Olla, Ismael Fernandez) who took two days off from touring with Madonna to visit the GRC and underscored the extreme complexities of skilled movement.

Looking to the future, a second GRC on Parkinson’s Disease has been scheduled for the summer of 2017 and will be chaired by Dr Singleton and co-chaired by Tim Greenamyre, MD, PhD. After this, the Gordon Research Conferences staff will determine whether to add the GRC on Parkinson’s Disease into the regular rotation of meetings. If this happens, there will be a GRC on PD every 2 years. Keep your fingers crossed, because the addition of this meeting is likely to revolutionize the field of Parkinson’s disease research.

